# A Case of a Combined Distal Radius and Lunate Fracture in Association With Kienböck’s Disease

**DOI:** 10.7759/cureus.31161

**Published:** 2022-11-06

**Authors:** David O'Sullivan, Patrick Carroll, John Galbraith

**Affiliations:** 1 Department of Trauma and Orthopaedics, Galway University Hospitals, Galway, IRL

**Keywords:** scaphocapitate fusion, salvage surgery, internal fixator, distal radial fractures, kienböck’s disease

## Abstract

The natural history of Kienböck’s disease (KD) is often indolent until it progresses to an advanced stage causing pain and stiffness. Lunate fragmentation and collapse can sometimes occur with trauma. However, concomitant fracture of the distal radius is a rare phenomenon, and this combination can limit treatment options. The aim of this case report is to outline our management algorithm in the operative fixation of a young mechanic with known ipsilateral KD who was involved in a road traffic accident and suffered a combined distal radius and lunate fracture. He was managed with an open reduction internal fixation using a volar plate and a temporary dorsal spanning internal fixator. At a later date, the internal fixator was removed and a scaphocapitate fusion was performed to offload the lunate in order to avoid a total wrist fusion.

## Introduction

Kienböck’s disease (KD) is an osseous condition characterised by avascular necrosis of the lunate. The condition is a spectrum, and pathology ranges from asymptomatic incidental altered signal on T1-weighted magnetic resonance imaging (MRI) to atraumatic wrist pain and lunate collapse. Patients often remain asymptomatic until advanced disease progression, where patients begin to complain of wrist pain, instability, and decreased grip strength. The diagnosis of KD can be made through plain radiographs; however, in order to thoroughly assess lunate viability and the degree of osteonecrosis, an MRI and wrist arthroscopy are often utilised [1]. The management of patients diagnosed with KD is challenging. The biomechanical principle behind the surgical management of KD is to offload the lunate to protect its viability or bypass the lunate when deemed not salvageable. The objective is to preserve wrist stability and function, especially in the younger cohort of patients [2]. A concomitant distal radial fracture in conjunction with KD is a rare phenomenon which presents a number of technical challenges [3]. This report describes the operative algorithm employed to manage the dual pathology.

## Case presentation

**Figure 1:**
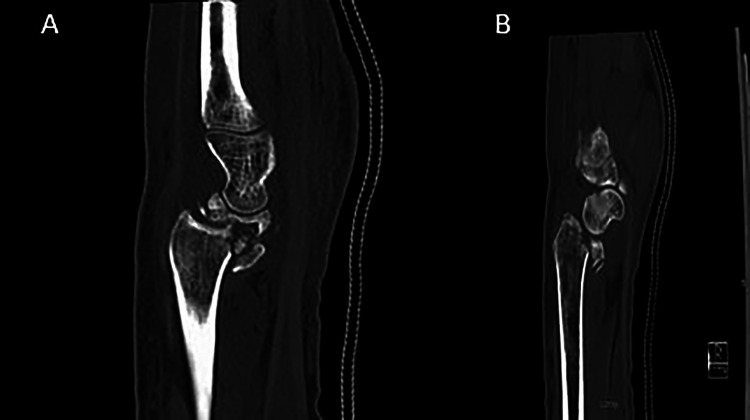
(A and B) Sagittal slices of the computed tomography image outlining the incongruity of the scaphoid fossa. Figure 1A also outlines the collapse of the lunate.

A 25-year-old right hand dominant mechanic was referred to our tertiary referral centre after a road traffic accident (RTA) with a comminuted, volarly displaced fracture dislocation of his left distal radius combined with a comminuted lunate fracture (Figure 1). The patient had a known diagnosis of ipsilateral KD. The injury was closed, and on examination the patient’s neurovasacular status was intact. A computed tomography (CT) scan demonstrated a distal fracture with incongruity of the scaphoid fossa (Figure 1).

**Figure 2:**
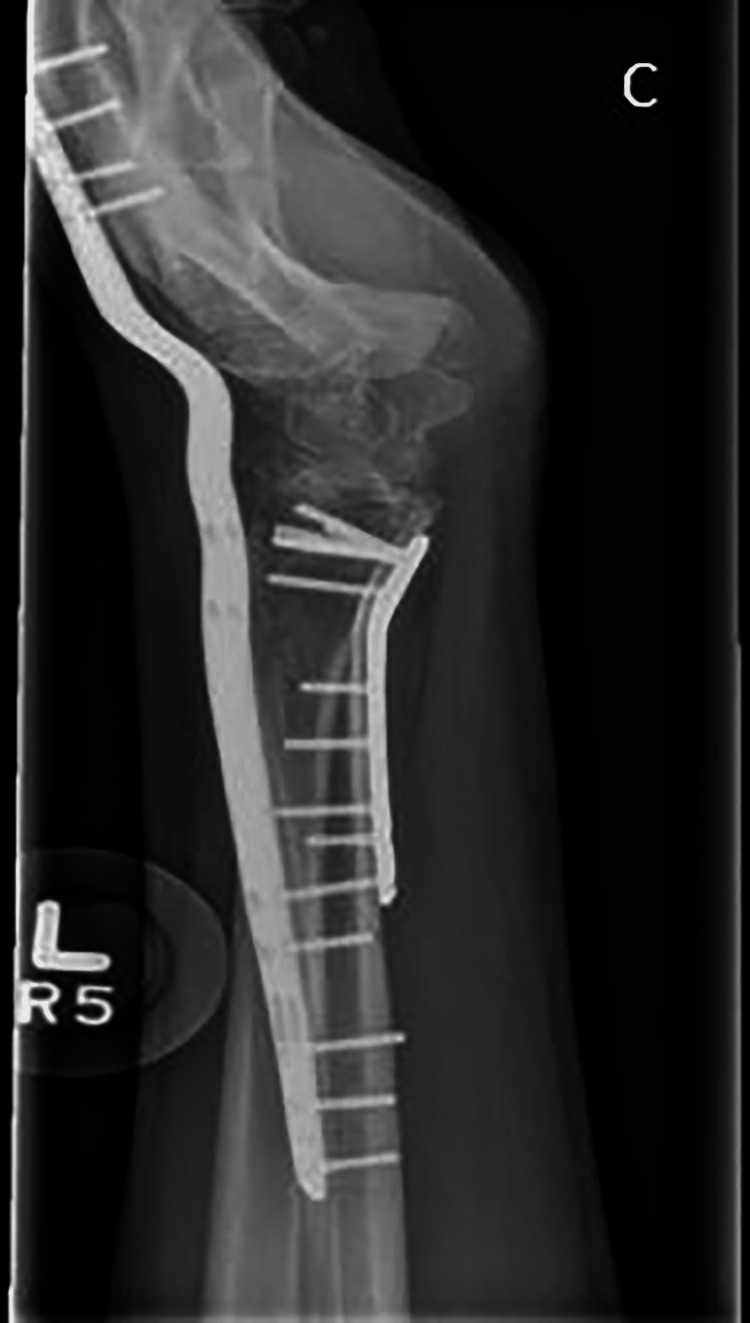
Lateral radiograph after the first stage of surgery. Length alignment and rotation has been resorted through the volar plate and dorsal fixator.

The lunate was non-reconstructable, and any future salvage procedure would require a congruent radioscaphoid joint. The proximity of the fracture to the radioscaphoid joint meant there was concern that volar plate fixation alone would be insufficient to maintain joint congruity. Therefore, a staged approach was taken. Initially, a volar locking plate fixation (Medartis, Basel, Switzerland) was used to reduce the scaphoid fossa. In addition, a dorsal wrist-spanning plate (Medartis) was applied to offload the volar fixation (Figure 2). The dorsal spanning plate was removed eight weeks post-operatively, and range-of-motion (ROM) exercises were commenced.

Five months after the initial injury, a scaphocapitate fusion (SCF) was performed as a salvage procedure. A dorsal approach was utilised, the scaphoid and capitate were decorticated and bone graft was taken from the dorsal aspect of the ipsilateral radius. Two 3-mm screws (Medartis) were used to achieve fusion. Post-operative radiographs showed satisfactory alignment of the arthrodesis and unity of the previous distal radial fracture fixation.

**Figure 3:**
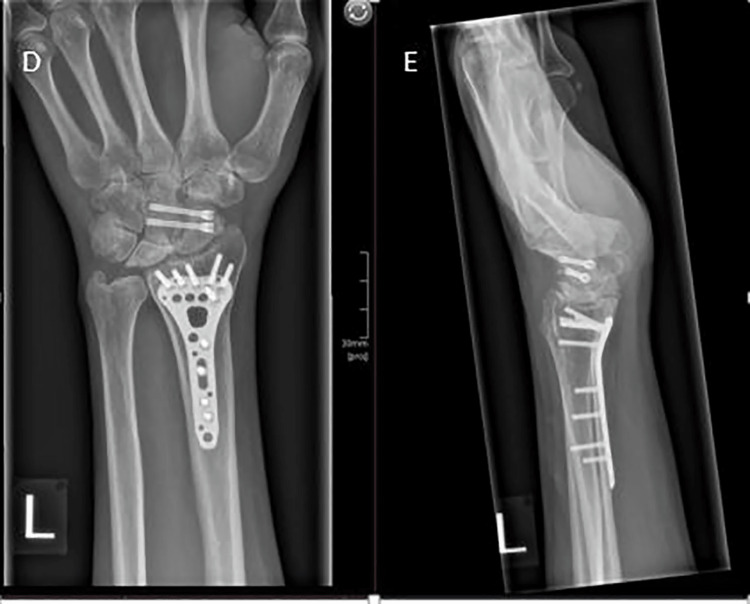
Post-operative anteroposterior (D) and lateral (E) radiographs showing the placement of the SCF screws, fusion of the scaphoid and capitate, and a healed distal radius fracture. SCF, scaphocapitate fusion

At one-year follow-up from the index injury and seven months post-SCF, the patient reported a pain-free wrist with good function. Wrist ROM was 20 degrees in flexion and 40 degrees in extension. Final imaging demonstrated union of his SCF and preservation of the radioscaphoid joint (Figure 3).

## Discussion

Combined distal radius and lunate fracture is a rare diagnosis, with the literature base limited to a handful of case reports [3]. The above injury pattern has not previously been described in the literature.

This was a complex case that required a staged approach to definitively treat the patient’s pathology. The concurrent presence of KD associated with a distal radius fracture dislocation necessitated increased stability initially at the fracture site and radiocarpal joint. A combined fixation technique utilising a volar plate and dorsal spanning fixator has previously been described as a management option for comminuted, unstable distal radial fractures [4]. The internal spanning fixator establishes ligamentotaxis, which temporarily restores length and alignment, and is used in conjunction with volar plate; the dorsal spanning plate provides a buttress effect and reduces the likelihood of collapse [5].

The current treatment algorithm, proposed by Lichtman et al. for the management of KD, combines multimodal imaging, arthroscopy, and patient factors to aid surgeons in their preoperative planning and provides an evidence-based approach for the management of KD [2]. SCF is a surgical technique indicated for the management of KD in the presence of a fractured lunate or in the presence of carpal collapse (stage IIIB-IV) [1]. Biomechanically, an SCF has been shown to shift the force transferred from the hand and wrist through the radiolunate to the radioscaphoid joint and thus theoretically reduce symptoms associated with KD [6]. The procedure has also been shown to decrease pain and improve quality of life in patients with advanced KD [7]. In our patient, the scaphoid fossa of the radius was fractured and would not have supported a lunate offloading procedure. Rather than proceeding to an acute total wrist fusion, a decision was made to reconstruct the scaphoid fossa of the radius to permit a delayed SCF. In view of the age of the patient and his occupation as a mechanic, some preservation of wrist ROM was desirable. The volar fixation was tenuous owing to the comminution and the proximity of the fracture to the joint line. The dorsal spanning plate offloaded the distal radius, thus protecting the volar fixation from early failure. The ROM was reduced at the final follow-up; however, the presence of an intact radioscaphoid joint makes a soft tissue release procedure a viable option in the future.

## Conclusions

In conclusion, this is a rare presentation of a concomitant fracture dislocation of the distal radius and lunate in a patient with ipsilateral KD. This patient underwent both volar and dorsal fixation, as well as an SCF as a salvage procedure. This injury pattern is rare, and patients with such injuries need to be counselled prior to the index procedure on reduced functional outcomes.
